# Possible Pet-associated Baylisascariasis in Child, Canada

**DOI:** 10.3201/eid1802.110674

**Published:** 2012-02

**Authors:** Shariq Haider, Krishna Khairnar, Donald S. Martin, James Yang, Filip Ralevski, Kevin R. Kazacos, Dylan R. Pillai

**Affiliations:** McMaster University, Hamilton, Ontario, Canada (S. Haider);; Ontario Agency for Health Protection and Promotion, Toronto, Ontario, Canada (K. Khairnar, F. Ralevski, D.R. Pillai);; IDEXX Reference Laboratories Ltd., Markham, Ontario, Canada (D.S. Martin, J. Yang);; Purdue University, West Lafayette, Indiana, USA (K.R. Kazacos);; University of Toronto, Toronto (D.R. Pillai)

**Keywords:** baylisascariasis, Baylisascaris procyonis, parasites, raccoons, humans, Canada, encephalitis, roundworms

**To the Editor:** *Baylisascaris procyonis*, a roundworm parasite of raccoons (*Procyon lotor*), increasingly is being documented as a cause of severe human disease (*1*). Approximately 130 species of wild and domesticated animals have been affected with *B. procyonis* neural larva migrans, and the parasite is increasingly recognized as a cause of human encephalitis (*2*; K. Kazacos, unpub. data). The first recognized human case was reported in 1984 in a 10-month-old child in Pennsylvania, USA (*3*). Since then, ≈30 additional cases of severe or fatal *B. procyonis* encephalitis have been reported in the United States (*4**–**7*; K. Kazacos, pers. comm.). To our knowledge, only 1 account of human *B. procyonis* infection has been reported in Canada (in 2009) (*8*). We report another case of human *B. procyonis* infection in Canada, indicating its probable transmission from peridomestic raccoons.

In 2008, a 14-month-old previously healthy boy in Hamilton, Ontario, Canada, sought care for fever, regression in speech for 5 days, and failure to bear weight for 2 days. His parents also noticed that he was not tracking with his eyes. Caregivers recalled a macular rash on the face and trunk that had faded over time. The child was hospitalized, and a workup for encephalitis was initiated. He was hemodynamically stable and had flaccid tone, with inability to bear weight. No visible rashes were found. A fundoscopic examination indicated no evidence of unilateral chorioretinitis. The child was unable to track objects, which suggested vision loss in both eyes. Blood cultures, urine cultures, and lumbar puncture were performed. Results of blood analyses showed the following: lymphocytes 24% (45%–76%), monocytes 41% (3%–6%), eosinophils 32% (0%–3%), protein 34 g/L (42–74 g/L), and glucose 3.0 mmol/L (3.3–5.8 mmol/L). Magnetic resonance imaging of the brain showed diffuse white matter lesions scattered in the subcortical and deep white matter over both cerebral hemispheres and periventricular region, most prominent in the left parietal lobe and frontoparietal regions, and subtle hyperintense lesions in bilateral dentate nucleus (Figure, panel A). No meningeal enhancement was noted.

**Figure:**
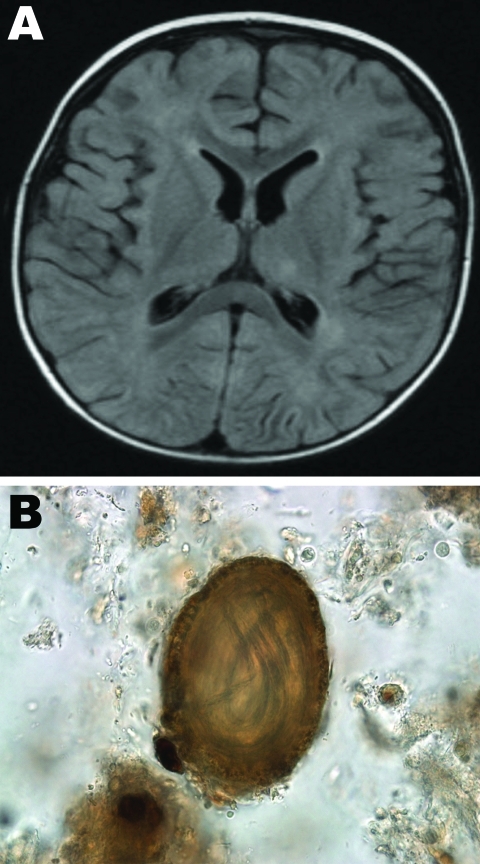
A) Magnetic resonance imaging of the brain of a 14-month-old child with baylisascariasis encephalitis. B) *Baylisascarasis procyonis* embryonated egg found in wet preparation of raccoon feces; original magnifi cation ×100.

Because of eosinophilic meningoencephalitis, thorough analyses were conducted for immunologic and infectious etiologies. The family confirmed the presence of numerous raccoons in their backyard, which raised a concern for *Baylisascaris* encephalitis, and samples of cerebrospinal fluid and serum were sent to Purdue University (West Lafayette, IN, USA) for serologic testing. On the basis of clinical findings, the child was given albendazole 200 mg orally 3×/day and prednisone 25 mg orally for 4 weeks. Results of ELISA were positive for *B. procyonis* from serum (optical density = 0.744; cutoff 0.250) but negative from cerebrospinal fluid. *B. procyonis*–specific protein bands were seen on Western blotting (*9*). The parents reported that the child had no access to the backyard, but they and their dog often moved between the backyard and the house. Environmental sampling was conducted in conjunction with the public health department. Thirty samples were taken from the patient’s home and yard. A sample of raccoon feces taken from a garbage bag from the porch of the house contained embryonated *B. procyonis* eggs (Figure, panel B). No eggs were identified in the dog. Nine months after the initial hospitalization, the child had substantial physical and motor delays, was legally blind in both eyes, and had epilepsy.

To our knowledge, this is the second case of *Baylisascaris* encephalitis identified in Canada. Both cases are noteworthy for profound neurologic impairment. Similar to cases reported from the United States, the case reported here highlights the dangers of peridomestic raccoons, which are becoming increasingly common in both countries. Although the classical risk factors for pica/geophagia or developmental delay were not reported by the patient’s parents, he could have become infected only through ingestion of infective eggs, from an as-yet-undetermined location, object, or source. The case reported here illustrates the need for a collaborative approach in unusual cases; we included clinicians and public health and laboratory specialists in the workup of this case. We found a strong correlation between the serologic findings, the child’s clinical signs, other clinical information (e.g., eosinophilia, magnetic resonance imaging findings), the age of the child, and the recovery of embryonated *B. procyonis* eggs from his environment. We postulate that he became infected by ingesting raccoon feces/infective eggs unintentionally brought into the home or through exposure in adjacent structures, such as the porch. Although no eggs were identified in the dog, several reports have documented intestinal infection of domestic dogs with *B. procyonis*, albeit at a prevalence thousands of times lower than that in raccoons (*3*; J. Yang, unpub. data). That the dog served as a vector after being exposed to raccoon feces and infective eggs is far less likely. A recent report from the Centers for Disease Control and Prevention suggests possible transmission from pet kinkajous (*10*). We speculate that more common domestic pets also might serve as possible reservoirs for and sources of infection.
